# Value Iteration Networks with Double Estimator for Planetary Rover Path Planning

**DOI:** 10.3390/s21248418

**Published:** 2021-12-16

**Authors:** Xiang Jin, Wei Lan, Tianlin Wang, Pengyao Yu

**Affiliations:** School of Naval Architecture and Ocean Engineering, Dalian Maritime University, Dalian 116026, China; jinxiang@dlmu.edu.cn (X.J.); lanwei@dlmu.edu.cn (W.L.)

**Keywords:** planetary rover path planning, reinforcement learning, value iteration algorithm, deep neural network, double estimator method

## Abstract

Path planning technology is significant for planetary rovers that perform exploration missions in unfamiliar environments. In this work, we propose a novel global path planning algorithm, based on the value iteration network (VIN), which is embedded within a differentiable planning module, built on the value iteration (VI) algorithm, and has emerged as an effective method to learn to plan. Despite the capability of learning environment dynamics and performing long-range reasoning, the VIN suffers from several limitations, including sensitivity to initialization and poor performance in large-scale domains. We introduce the double value iteration network (dVIN), which decouples action selection and value estimation in the VI module, using the weighted double estimator method to approximate the maximum expected value, instead of maximizing over the estimated action value. We have devised a simple, yet effective, two-stage training strategy for VI-based models to address the problem of high computational cost and poor performance in large-size domains. We evaluate the dVIN on planning problems in grid-world domains and realistic datasets, generated from terrain images of a moon landscape. We show that our dVIN empirically outperforms the baseline methods and generalize better to large-scale environments.

## 1. Introduction

The planetary rover can move freely on the planet’s surface and conduct exploration missions over a larger area than a fixed lander. However, given the distance from Earth and the communication delay, it is difficult for the rover to receive real-time control commands from the ground to guide its exploration activities. When the rover needs to go a target out of view, planning a collision-free and energy-efficient path is challenging because the uncertain planetary environments and the limited detection range of the sensors on board make it more vulnerable to hazards, such as rocks, slopes, and craters [1].

One possible solution is to calculate the globally optimal path in advance, based on the high-resolution terrain images taken by satellites, and then let the rover follow this path based on the actual observation. Search-based planning algorithms [2], including the popular Dijkstra and A*, are common approaches to path planning problems and are guaranteed to find a solution path, if one exists, by incrementally exploring the map. PRM and RRT are examples of sampling-based planning algorithms [3], which randomly sample the configuration space to create a path. In high-dimension planning problems, the sampling-based algorithms work more efficiently than the search-based methods, in terms of computational resources, but at the cost of being non-optimal. Heuristic-based approaches [4], e.g., genetic algorithm (GA) and particle swarm optimization (PSO), have high proficiency in handling unknown or partially known environments.They create a set of temporary paths within each of their iterations and choose the best path, based on their fitness, gradually bringing them closer to the destination location. However, these classical path planning algorithms [5] often require mapping the environmental information to grid maps or Voronoi diagrams and can not work without additional assumptions or human’s prior knowledge about planetary environments [6]. They can only search for an optimal policy for an instance of a certain type of environment at a time, and the policy could not generalize to other similar, but unseen, domains.

Deep reinforcement learning (RL) aims to address this issue by first using deep neural networks(DNNs) to extract environmental features, and then use RL algorithms to plan, with respect to the learned features [7,8]. Model-free RL methods have devoted to directly estimating the optimal policy or value function from massive interactions with the environment [9,10], where the DNN is usually similar to that employed in supervised learning. Despite a lack of explicit planning, the learned policy is effective because RL algorithms themselves have access to high, long-term returns. While model-based RL methods have focused on reconstructing a model of the environment’s dynamics from the sequential inputs of full observations and then planning in the learned model [11,12], which can effectively generalize to novel tasks.

Instead of explicitly constructing environment models and performing planning algorithms, the value iteration network (VIN) has been proposed to combine the advantages of model-free and -based methods, using the recurrent convolution layers to approximate the value iteration (VI) algorithm and realizing end-to-end planning, without pre-defined environment’s dynamics and reward functions [13]. While the VIN can only take regularly structured data as input and the Markov decision process (MDP) behind must be fixed and known, some recent works have further extended the usage of the VI-based architecture to irregular graphs [14,15], dynamic environments [16], and spatially variant problems [17].

VI-based models are often plagued by training instability and poor convergence because of the stacked convolution and max-pooling layers in the VI module. The modern practice of convolutional neural networks has shown that the deeper networks are more expressive. However, when the depth reaches a certain level, the network is difficult to train effectively; not only does convergence becomes slower, but the performance even decreases. In practice, deep neural networks have widely used (1) residual structures [18] to solve the problem of vanishing/exploding gradients and (2) normalization operations [19] to stabilize the training process and increase the generalization ability. However, we experimentally demonstrate that these techniques are not helpful in training for stability and accuracy improvement of VIN.

In this work, we present a variant of VIN, termed a double value iteration network (dVIN). This model implicitly learns to use training data from other planners to solve planning problems of planetary rovers and relies only on orbital images to produce a plan that is useful in situations beyond the visible range of the ground. Inspired by [20,21], we introduce two estimators to the VI module, decoupling the action selection from evaluation and propose a new VI-based model. This double estimator method prefers overestimated to underestimated values, resulting in more stable and reliable learning, and the alternate update of two estimators acts as a regularizer, having a beneficial effect on the gradient flow through the network.

**Figure 1:**
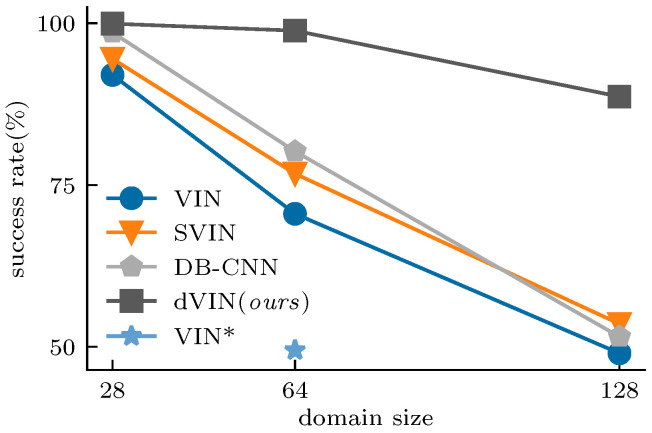
Success rate in test set vs. domain size. Our dVIN outperforms three baseline methods on the grid-world path planning task, especially on large-size domains. VIN* is trained from scratch in 64×64 domains. Additionally, its performance is much lower than that of the VIN trained with the two-stage training strategy.

In addition, the resolution of feature maps keeps fixed in the VI-based model, leading to a high computational cost, especially in large-sized environments. However, we found the VI-based model has good transfer capability among different size domains by adjusting the iteration number of VI according to the input resolution. Therefore, we design a two-stage training approach for VI-based models. The resulting model has much better performance than that trained from scratch on large-size domains, as shown in Figure 1.

We demonstrate that, when combining the double estimator method with a two-stage training strategy, the dVIN significantly improves the performance of VIN on path planning tasks and outperforms the CNN-based model [22] by a wide margin in large-size environments. The main contributions of this work are summarized as follows:

- By introducing a double estimator approach, we propose the double value iteration network, a variant of the VIN that can effectively learn to plan from natural images.
- We design a two-stage training strategy, based on the characteristics of VI-based models, which can help them achieve a better performance in large-size environments with a lower computational cost.
- Experimental results on grid-world maps and terrain images show that the dVIN significantly outperforms not only the previous VI-based models but also a CNN-based model.

The paper is organized as follows. Section 2 provides some preliminaries of this paper. Section 3 describes the proposed dVIN architecture for global path planning. The experimental results and some discussion are presented in Section 4. The conclusion is given in Section 5.

## 2. Preliminaries

### 2.1. Markov Decision Processes

The MDP can usually be represented as a tuple $\left[S,A,P,R\right]$, with S being the set of state *s*, A being the set of action *a*, *P* the state-transition distribution mapping each state-action tuple $\left(s,a\right)$ to a probability distribution over states (with $p\left(s^{\prime }|s,a\right)$ denoting the probability of transitioning to state $s^{\prime }$ from *s* by choosing action *a*) and R⊂R the set of reward *r*. In RL, the goal of the agent is to select actions in a fashion that maximizes the cumulative expected reward.

A *policy* π is a mapping from each state to a distribution over actions (with $\pi \left(a|s\right)$), denoting the probability of choosing *a* in state *s*). If the current state is $S_{t}=s$ at time *t*, and actions are selected according to a stochastic policy π, the *value function*, denoted $v_{\pi }\left(s\right)$, is the expected return when starting in *s*, following π thereafter, and can be defined by:

(1) $$v_{\pi }\left(s\right)=E_{\pi }\left[\sum_{k=0}^{}\gamma^{k}R_{t+k+1}|S_{t}=s\right],$$

where $\gamma \in \left[0,1\right]$ is a discount factor that trades off the importance of immediate and later rewards.

Similarly, we define the *action-value function* as the expected return, starting from *s*, taking the action *a*, and thereafter following policy π, which can be written as:

(2) $$q_{\pi }\left(s,a\right)=E_{\pi }\left[\sum_{k=0}^{}\gamma^{k}R_{t+k+1}|S_{t}=s,A_{t}=a\right].$$

We denote all the optimal policies by $\pi_{*}$, and they share the same state-value function, called the *optimal state-value function*, denoted $v_{*}$ and defined as $v_{*}\left(s\right)=\max_{\pi }v_{\pi }\left(s\right)$. The *optimal action-value function* $q_{*}$ is defined as $q_{*}\left(s,a\right)=\max_{\pi }q_{\pi }\left(s,a\right)$. We can further write $q_{*}$, in terms of $v_{*}$, as:

(3) $$q_{*}\left(s,a\right)=E\left[R_{t+1}+\gamma v_{*}\left(S_{t+1}\right)|S_{t}=s,A_{t}=a\right].$$

The Bellman optimality equation expresses the fact that the value of a state under an optimal policy must equal the expected return for the best action from that state, that is $v_{*}\left(s\right)=\max_{a}q_{\pi_{*}}\left(s,a\right)$. An effective way to finding an optimal policy is *value iteration*:

(4) $$v_{k+1}\left(s\right)=\max_{a}\sum_{s^{\prime },r}^{}p\left(s^{\prime },r|s,a\right)\left[r+\gamma v_{k}\left(s^{\prime }\right)\right].$$

For arbitrary $v_{0}$, $v_{k}$ can be shown to converge as k→∞ to $v_{*}$, under the same conditions that guarantee the existence of $v_{*}$ [8].

### 2.2. Double Q-Learning

Q-learning, a popular reinforcement learning algorithm, has successfully been used to optimally solve MDPs. The update of Q-learning can be given as:

(5) $$Q_{t+1}\left(s,a\right)=Q_{t}\left(s,a\right)+\alpha_{t}\left(s,a\right)\left(Y_{t}^{Q}-Q_{t}\left(s,a\right)\right),$$

where the target $Y_{t}^{Q}=r+\gamma \max_{a}Q_{t}\left(s^{\prime },a\right)$.

Q-learning approximates the value of the next state by maximizing over the estimated action values in that state. The max operator uses the same Q functions to select action and evaluate its value, which makes it more likely to select overestimated values, resulting in overoptimistic value estimates and a large performance penalty in stochastic MDPs. To avoid this overestimation, a double estimator method was proposed to decouple the selection operator from the evaluation.

A double Q-learing algorithm [20] stores two Q functions, and each function is updated with a value from the other for next state. Despite two set of weights, one of them is used to determine the greedy policy, with the other determining its value. The two functions are learned by randomly assigning each experience to update one of them, so the method is not less sample-efficient than standard Q-learning.

In [21], without having to introduce another set of weights, the target network in the DQN provides a natural candidate for the second Q function. Therefore, the online network is used to evaluate the greedy policy, but the target network is to estimate the value. The target can be written as:

(6) $$Y_{t}^{DDQN}=r+\gamma Q\left(s^{\prime },\underset{a}{\mathrm{argmax}}Q\left(s^{\prime },a;\theta_{t}\right);\theta_{t}^{-}\right).$$

Compared with double Q-learning, the weights of target network $\theta_{t}^{-}$, instead of the weights of a second network $\theta_{t}^{\prime }$, are used for the evaluation of the current greedy policy.

### 2.3. Value Iteration Network

Considering the planning problem in 2D grid-word domains, the purpose is to find a shortest path from the start position to the goal, without encountering any obstacles. Let *M* denote the MDP of the domain where we design our policy π. We also assume there is another unknown MDP $\bar{M}$, such that the optimal policy ${\bar{\pi }}^{*}$ contains some useful information that can be used to solve $\pi^{*}$ in the original MDP.

The VIN is an end-to-end architecture that is equipped with the ability to learn and solve $\bar{M}$ and add the solution as a part of the policy π. To establish a connection between *M* and $\bar{M}$, we let $\bar{r}=f_{R}\left(\phi \left(s\right)\right)$ and $\bar{p}=f_{P}\left(\phi \left(s\right)\right)$, where $\phi \left(s\right)$ is denoted by the observation of the state *s*, and the functions $f_{R}$ and $f_{P}$ will be as some elements in the policy learning process.

We further assume that $\bar{M}$ has the same state and action spaces as *M*, namely, $\bar{s}=s$, $\bar{a}=a$. Additionally, $f_{R}$ maps an image of the grid-world domain to a positive reward at the goal position, while $f_{P}$ encodes deterministic movements in the grid-world that the agent can once move from the current position to its neighborhood. The VI module can be approximately written as:

(7) $${\bar{v}}^{k}=\max_{a}{\bar{q}}^{k}=\max_{a}h\left(\bar{r},{\bar{v}}^{k-1}\right),$$

where $k\in \left[1,K\right]$ is the iteration number of the VI module.

In 2D navigation tasks, the MDP has the local connectivity structure. Therefore, the transition function *h* can be represented as a convolution operator. The VI policy can then be generally written as:

(8) $${\bar{v}}_{i,j}^{k}=\max_{a}{\bar{q}}_{a,i,j}^{k},\;{\bar{q}}_{a,i,j}^{k}=\sum_{\left(i^{\prime },j^{\prime }\right)}^{}W_{a,i^{\prime }-i,j^{\prime }-j}^{\left[r,v\right]}\left[{\bar{r}}_{i^{\prime },j^{\prime }},{\bar{v}}_{i^{\prime },j^{\prime }}^{k-1}\right],$$

with the tuple $\left(i^{\prime },j^{\prime }\right)\in N\left(i,j\right)$ being the neighbor of the agent’s position (i,j) and *W* the parameters of the convolution kernel.

**Figure 2:**
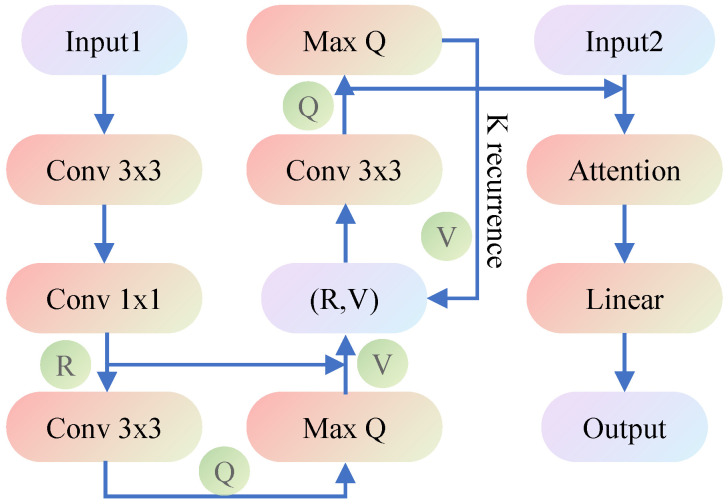
Architecture of the VIN. Input1is a $\left(2\times m\times n\right)$-sized image, where *m* and *n* represent the length and width of the domain, encoding the obstacle presence and goal position, respectively. Input2 is a tuple $\left(i,j\right)$ of the current position. Output is the probability distribution over available actions. The attention module is given to improve performance, by reducing the effective number of parameters during learning. The recurrence *K* was chosen, in proportion to the domain size, to ensure that information can flow from the goal position to any other position.

The architecture of VIN is depicted in Figure 2.

## 3. Methods

Each iteration of the VI module can be seen as passing through a convolution and max-pooling layer, and recurrently applying a convolution layer that is *K* times equivalent to the information flowing through a *K*-layer plain convolutional network. The expressive capacity of neural networks typically grows significantly as the depth increases, enabling strong generalization performance. It is necessary for the VIN because the depth should be efficient for the reward signal to propagate from the goal to any other position and be some function of the length of the shortest path.

Deeper networks are usually more difficult to train, resulting from vanishing/ exploding gradients and poor signal propagation. These problems have been largely addressed by residual learning [18,23], careful initialization, and normalization, such as BatchNorm and LayerNorm [24]. Practitioners have also proposed some similar methods to improve the performance of VIN, including sharing weights between layers, replacing the max-pooling operator with the softmax function [25] or intrducing a LSTM cell with complex gating mechanisms, instead of the convolution layer [26]. However, these methods either bring slight improvement in performance or have excessive computational cost.

The max-pooling operator in the VI module of Equation (8) can be simply rewritten as:

(9) $$V^{k}\left(s\right)=Q^{k}\left(s,\underset{a}{\mathrm{argmax}}Q^{k}\left(s,a\right)\right),$$

which includes a maximization step over estimated action values, which tends to prefer overestimating values and sometimes learns unrealistically high values. If overestimation occurs, the iterative algorithm can lead to overestimation propagating throughout our estimates, resulting in all the values being overestimated. Although this uniform overoptimism might not hurt the resulting policy, in practice, overestimation errors will differ, due to the approximate VI algorithm.

With the single estimator in Equation (9) resulting in the overestimation, we construct the two Q functions, $Q_{A}$ and $Q_{B}$, with same architecture but different parameters. While each value function is updated with the maximum valued action, according to one Q function, the estimated value of this action varies from the other, which can be expressed as:

(10) $$\begin{array}{cc} V_{A}^{k}\left(s\right) & =Q_{A}^{k}\left(s,\underset{a}{\mathrm{argmax}}Q_{B}^{k}\left(s,a\right)\right),\\ V_{B}^{k}\left(s\right) & =Q_{B}^{k}\left(s,\underset{a}{\mathrm{argmax}}Q_{A}^{k}\left(s,a\right)\right). \end{array}$$

The two estimates are then used as the maximum expected value, with the next iteration continued.

**Figure 3:**
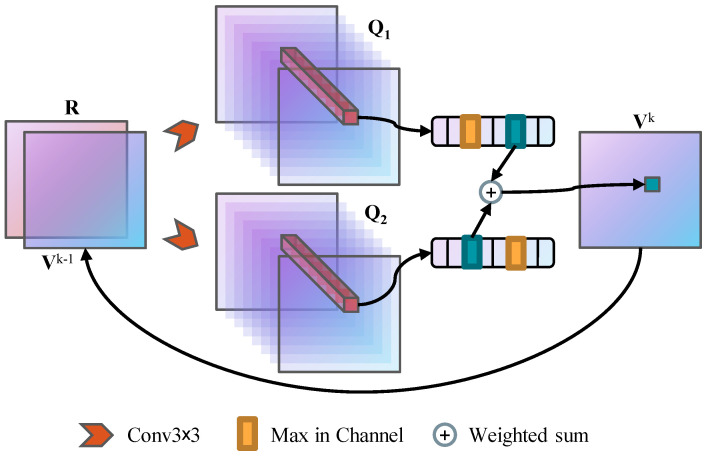
The approximate VI module with weighted double estimator.

In addition, ensemble methods are commonly used, in practice, to improve the performance of deep learning models [27,28]. Even by simply averaging the output of multiple weak learners, we can obtain a new model with better performance [29]. Ideally, if our model can be trained sufficiently, both estimators should be optimal, and either estimate can be used as the maximum expected value for the next iteration. However, in practice, two estimators are weak, so we use the ensemble of estimates as the maximum expectation for better performance. The estimated value can be written as:

(11) $$V^{k}\left(s\right)=\omega_{A}V_{A}^{k}\left(s\right)+\omega_{B}V_{B}^{k}\left(s\right),$$

where $\omega_{A}$ and $\omega_{B}$ are learnable scalar parameters, and $\omega_{A}+\omega_{B}=1$. Figure 3 shows the architecture of the weighted double VI module.

This double estimator method uncouples the selection of an estimator and its value, preventing the erroneously optimistic value function estimates and, instead, uses two estimates to learn a conservative estimate of the value function, providing a lower bound on the true value. Besides, this underestimation not only gives a clear motivation and interpretation, but contributes to facilitate well-behaved gradients and deep reward signals propagation, with these improvements being more noticeable in large-scale domains.

## 4. Experiments and Discussion

In this section, we evaluate the proposed dVIN architectures as policy representations of global path planning task in two kinds of environments, including synthetic grid-world domains and overhead terrain maps of a lunar landing site.

We compared our dVIN with three baseline methods, including VI- and CNN-based models, as follows.

- VIN: It is the first proposed NN architecture with explicit planning computation by end-to-end training. Our implementation follows most of the original settings in [30], using a 1×1 convolution layer to implement the reward function, 3×3 convolutional layer to approximate the state-transition function, and maximization operation, along the action channel, to obtain the state values.
- SVIN: Compared to the VIN, two optimizations have been proposed to enhance the network training and improve performance. One is to construct a multi-layer convolution network to produce better reward mapping, as in [30]. The second is to replace the max-pooling operation with the softmax of Q function over actions, under the assumption of probabilistic action policy, with the aim of more efficient gradient propagation. However, in this work, we focus on the performance of the VI module, so the multi-layered reward network is not considered in our implementation.
- DB-CNN: The task setting of path planning is more like semantic segmentation than image classification, assigning a semantic label to each pixel, which is the optimal action in our case. Ref. [22] designed a two-branch convolutional network, where branch 1 is similar to conventional CNNs for classification tasks, which extract global features by a series of stacked convolution layers and down-sampling operations, while branch 2 always keeps the feature map resolution consistent with the input for better local feature representation.

We empirically evaluate all models using the same metrics as [13], namely prediction loss, success rate, and trajectory difference. The prediction loss is defined as the average 0-1 loss of model outputs, relative to labels. A trajectory is said to be successful if it, by rolling out the models, reaches the target from the initial state without hitting any obstacle; the success rate measures the percentage of successful trajectories generated in test domains. The trajectory difference denotes the average deviation, in length, of the predicted successful ones from the optimal.

We also discuss the technical details of the model implementation and impact of training strategies on the model performance. All models and experimental code are implemented based on the PyTorch framework [31] and will be open-sourced in the future.

### 4.1. Grid-World Domain

Our first experimental domain is a grid-world with random placed obstacles. Each obstacle occupies one cell, and the number of obstacles increases with the domain size, but their percentage over all the available space is kept fixed for ease of difficulty control. The agent can take one step forward to eight adjacent cells at a time, with the aim of finding a collision-free shortest path from the start position to the goal.

#### 4.1.1. Performance in Small-Size Domains

On the one hand, because the baseline methods perform well on domains of size 8×8 and 16×16, it is difficult to bridge the performance gap between various methods. On the other hand, to directly compare the results reported in previous works, and effectively evaluate the quality of our baseline system, we start the first set of experiments on 28×28 domains.

The training set contains 10 k grid-world instances, where the obstacles are randomly generated, but the proportion of obstacles to the total available space is fixed at 50%. For each instance, we used the A* algorithm to calculate a shortest-path trajectory, so a total of about 10 k optimal trajectories were generated as training samples. The number is approximate because there may be no collision-free valid path between the random start and target positions.

We then represent each trajectory of length *T*, in the form of a state-action sequence $\left(s_{0},a_{0},s_{1},\ldots ,a_{T-1},s_{T}\right)$, where for each state-action pair $\left(s,a\right)$, the state $s=\left(i,j\right)$ denotes the x-y coordinate of current position, and the action *a* is an one-hot vector that denotes the best action corresponding to the state. A full training sample consists of a state-action pair $\left(s,a\right)$, plus an observation of the environment *o*, that can be represented as an image of size $\left(m\times n\times 2\right)$, with one channel encoding the obstacle presence (1 for obstacle, 0 otherwise), while the other encoding the goal position (1 at the goal, 0 otherwise), where $\left(m\times n\right)$ is the size of the domain.

We train each model for 30 epochs, with the RMSprop optimizer, and choose the initial learning rate of 0.004, with a mini-batch size of 128, while reducing the learning rate, by 10×, in the last 6 and last 2 epochs, respectively [32]. In addition, we also use the 1cycle learning rate scheduler [33], implemented in PyTorch, with the maximum learning rate of 0.008 and batch size of 256, which aims to alleviate the training instability of VINs, speed up the convergence, and improve the training accuracy. We refer to these two learning rate policies as steps LR and 1cycle LR, respectively. The recurrence *K* of the VI module is set to 1.5× the domain size; that is, K=42 for 28×28 domains.

**Figure 4:**
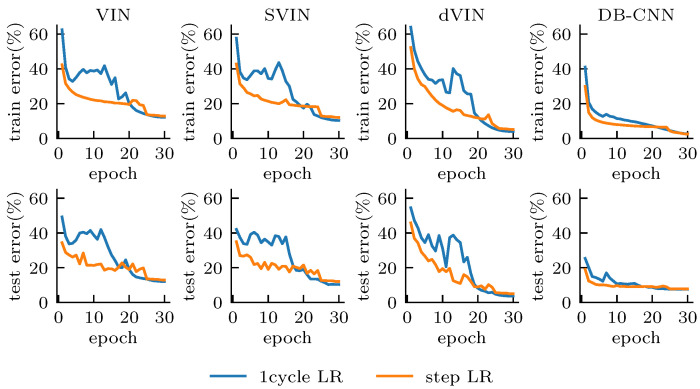
Training (**top**) and test error (**bottom**), with all models. The model trained with 1cycle LR converges better, although with a higher error in the early training stage. The dVIN has the best performance in the test set.

Figure 4 shows the training and test error for all models during training, and we can easily distinguish the differences between the CNN- and VI-based models from the curve features. The DB-CNN obtains a low error from the beginning of training, and the error always decreases steadily with almost no oscillation. These models are relatively insensitive to the choice of hyper-parameters and maintain a relatively stable performance overall for different random seeds and learning rate adjustment strategies, although 1cycle LR does lead to better accuracy, compared to step LR. Since the DB-CNN is essentially a reactive policy, lacking planning computation and mapping the state input to a one-step forward prediction, it performs weaker on the test set than on the training set, with 2.60% error on the training set under 1cycle LR, compared to 7.58% error on the test.

VI-based models, compared with the DB-CNN, seem to generalize better to domains outside the training set. The test and training error remain almost the same during training, indicating that they are not simply fitting the training set, but learning how to solve a class of problems through explicit planning computation. However, we also show that the VI-based models suffer from training instability and are more sensitive to the initialization conditions; choosing different random seeds can lead to large differences in the final performance. When adopting 1cycle LR, the VI-based models become more unstable in the early training stage. As the learning rate gradually increases to 2× the initial learning rate in step LR, the models almost stop converging, and the errors get saturated and oscillate at a high level; however, as the learning rate begins to decrease, the errors decrease rapidly and start to be smaller than that of using step LR at about 20 epochs and continues to decrease until the end of training.

We have observed that, after removing the deep reward network of SVIN, the softmax operation has limited improvement on model performance. In contrast, our proposed dVIN has a surprising performance, with much smaller error than other VI-based models on both the training and test sets. Although the training error is slightly higher than that of DB-CNN, the test error is only 3.28%, which is much lower than the 7.58% of DB-CNN, and even lower than the training error of 3.47%. Overall, our proposed dVIN architecture substantially improves the performance of VI-based models on path planning tasks and is also able to generalize better to new, unseen task instances, compared to the CNN-based model.

Table 1 shows more experimental results. It is worth noting that the success of a path is the result of successive multi-step decisions and does not only depend on the quality of the single-step prediction; so, there is no strict correspondence between the prediction loss and the success rate between different models, but, in general, the lower the prediction loss, the higher the final success rate. However, we also find that if two models have similar prediction losses, the simple model usually has a higher success rate, which is intuitive. Thus, although the dVIN is a bit more complex, compared to the vanilla VIN, its much lower prediction loss still guarantees a higher success rate than the latter.

Moreover, although the DB-CNN has smaller prediction loss and higher success rate than the VIN and SVIN, its trajectory difference is not much different from the latter two. Even when using 1cycle LR, the DB-CNN has a 5% higher success rate than the SVIN, but its trajectory difference is larger. This further indicates that, compared to the CNN-based model, the VI-based models is not limited to single-step prediction, but focuses more on long-term planning.

#### 4.1.2. Generalization in Large-Size Domains

Previous works have shown that the VIN performs well on small-size domains, thanks to the efficient propagation of the reward signal across the whole map, but as the map size increases, the VI module performs the planning computation for a long horizon by increasing the number of iterations. However, this not only increases the computational cost significantly, but also makes the unconstrained iterative process more unstable, making it difficult for the model to be trained effectively; thus, the performance on large-size maps degrades rapidly.

In fact, the state-transition distribution does not change when the domain size increases, so we further test how these models, trained on small-size maps, will perform on large maps. We can apply the VI-based model maps different sizes by adjusting the number of iterations *K*. However, with the original implementation, in [3], requiring a fixed input image size, we devised a new version, called DB-CNN*. We added an adaptive global average pooling layer on top of the last convolution layer, which pools the features and generates fixed-length output. We also referred to [34] and scaled up the model accordingly, so that the last convolution layer can output feature maps of size 4×4, with a 128×128 image fed into the network. Compared with the vanilla DB-CNN, the modified model has a similar performance on 28×28 domains.

We constructed two test sets, containing 5000 maps, with sizes of 64×64 and 128×128, respectively, where the percentage of obstacles to the whole space remains at 50%. Table 2 shows the performance of all models on these two data sets, and we emphasize that these models were only trained on 28×28 domains.

The VI-based models achieve better performance than the DB-CNN*, indicating that, even with the modified architecture, CNN-based models have difficulty generalizing to larger domains, due to different policy mechanisms. Specifically, the VIN achieves a success rate of 69.96% on 64×64 maps, 20% higher than that reported in [3], and the latter result was obtained by training the network for 100 epochs, using 10 K instances. Our dVIN achieves an even higher success rate of 87.65% on 64×64 maps, which is 7% higher than that of the DB-CNN trained from scratch, and 60.9% on 128×128 maps, which is also much higher than the other three methods. It is worth emphasizing that we trained all models on 28×28 maps only, requiring fewer computational resources than training models from scratch on larger size maps.

#### 4.1.3. Fine-Tuning in Large-Size Domains

We further consider whether we could achieve better results for VI-based models if we continued to fine-tune them with a small number of large-size instances. So, we constructed two small data sets, containing only 100 maps, with sizes of 64×64 and 128×128, for tuning the models well-trained on 28×28 maps. We trained each model for 10 epochs, with an initial learning rate of 0.0002, decaying the learning rate to 0.95 at the end of each epoch and setting a batch size of 32, considering the limitation of GPU memory.

**Figure 5:**
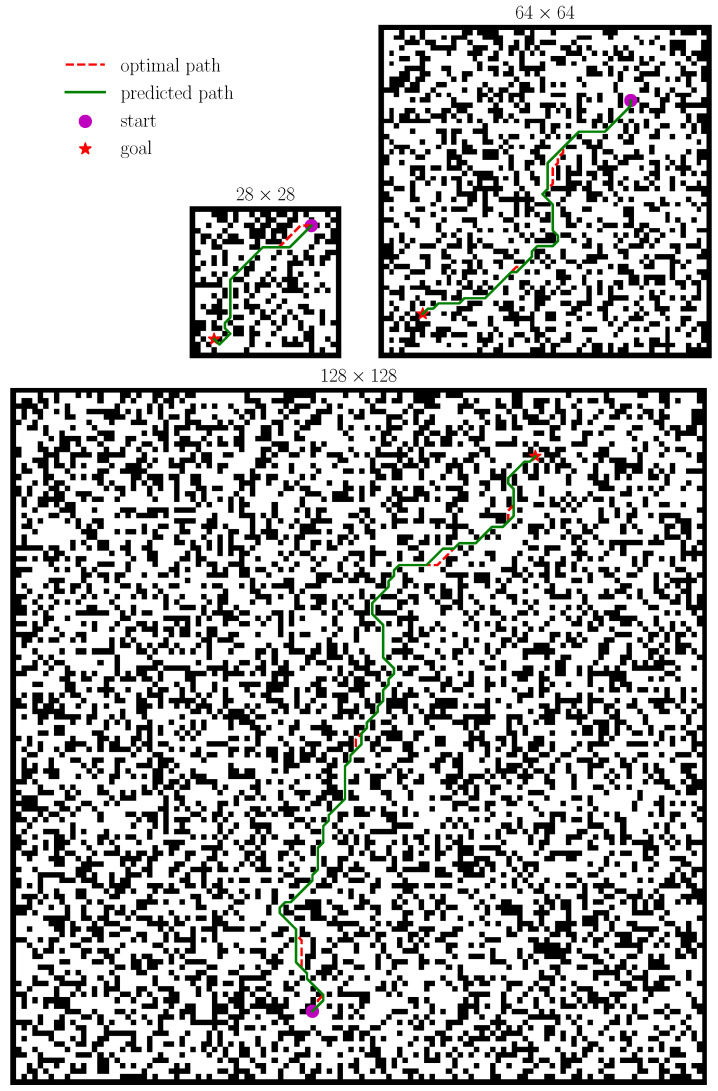
Random instances of grid-world domains with the size of 28×28, 64×64, and 128×128, with the dVIN-predicted trajectories and ground-truth shortest paths between random start and goal positions.

Figure 5 shows a set of grid-world instances with different sizes. Table 3 shows the performance of fine-tuned models on the test sets containing large-size maps.

We can see that VI-based models perform better than the DB-CNN, which has difficulty benefiting more from the two-stage training strategy. Specifically, the VIN and SVIN show limited improvement on 64 × 64 maps but have nearly 15% improvement, compared to the non-tuned model on 128 × 128 maps. The success rate of dVIN is even 98.85% on 64 × 64 maps, which indicates that our double estimator method substantially improves the upper-performance limit of the VI-based model and can outperform the CNN-based model, with less computational cost after training, with a simple two-stage training strategy. The dVIN also has a success rate of 88.66% on 128 × 128 maps, almost the same performance as the VIN and SVIN on 28 × 28 maps, which demonstrates that the relatively conservative value estimation method enables a more stable signal propagation on large-size domains and, thus, a better long-term behavior.

Overall, although path planning on grid-world domains seems to be a relatively simple task, NN-based methods usually perform poorly on large-size maps, as shown in Figure 1. Our proposed dVIN architecture benefits from the contrastive learning of two value estimators, avoiding the appearance of outliers during the iterations that may affect the stable propagation of the gradients. In addition, the two-stage training strategy of fully training on small-size maps and then fine-tuning on large-size maps, combined with the advantage of parameter sharing of VI-based models, helps the dVIN scales better with the size of the environment at a small cost.

### 4.2. Rovers Navigation

**Figure 6:**
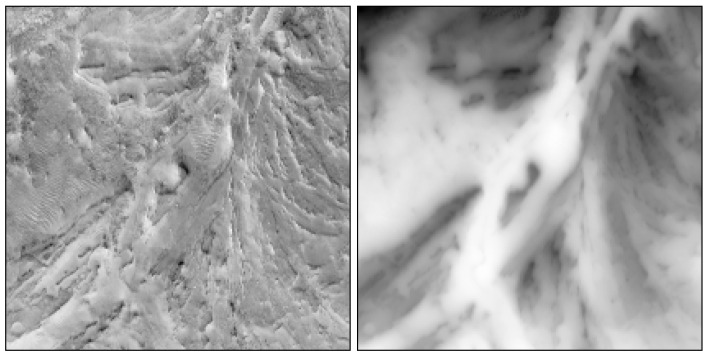
The orthomosaic (**left**) and corresponding DEM (**right**) of the Apollo 17 landing site, with a resolution of 0.5 m per pixel.

We further explore whether the dVIN can learn better planning strategies than the baseline methods, when taking natural images as input. We demonstrate this on the orthomosaic (overhead terrain images) of the Apollo 17 landing site, as shown in Figure 6, which has a resolution of 0.5 m per pixel, created from images provided by the Lunar Reconnaissance Orbiter Camera.

**Figure 7:**
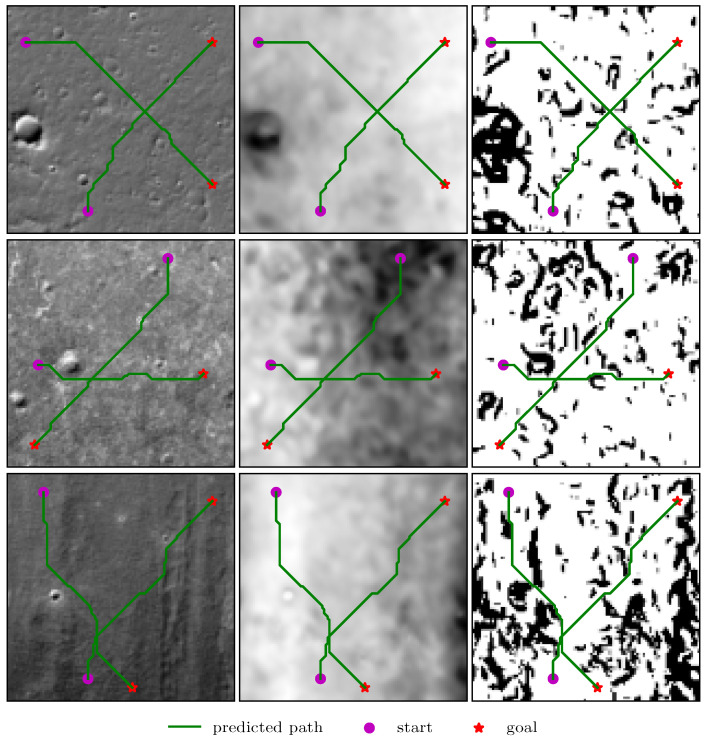
The predicted trajectories between random start and target positions in the lunar domains. From left to right: terrain image, DEM, and grid map, generated from DEM with the resolutions of 128×128. Note that the terrain image is used as the network input, and the elevation data is not available for the learning model.

We cropped the orthomosaic into non-overlapping image patches, as input to neural networks, and considered regions with an elevation angle of 10 degrees or more as obstacles, based on the external DEM data. We then randomly specified the start and target positions on obstacle maps and used the A* algorithm to generate the shortest paths as training samples. Figure 7 shows a cropped terrain image and the matching grid map, generated from the external elevation data. It is worth noting that the DEM data was only used to generate expert paths and is not part of the input to neural networks, which can only see terrain images and must infer elevation data from them.

The experimental setup remains the same as in the previous section, i.e., the models are fully trained on small-size images, and then fine-tuned on large-size ones. We do not include the CNN-based model in the comparison because the two-stage training strategy is not suitable for the DB-CNN, and it is too expensive to train models from scratch on large-size images.

Besides increasing the iteration number of the VI module, previous works usually added more layers to improve the expressive capability of models, when using higher resolution images as input [13,22,25]. However, our results show that we can obtain better performance without adding more network layers under the two-stage training method because increasing the number of iterations is somehow equivalent to stacking more layers, and deeper networks are usually better feature extractors with more expressive power. Additionally, due to the parameter sharing of the K recurrent layers, the VI-based models generalize better than the CNN-based model, while the effective depth is much larger.

The results in Table 4 show that the VI-based models can learn to plan on natural pictures and have an even higher planning success rate than in the grid-world domains, when the input resolution is the same. Here, we do not consider the difficulty of distinguishing between obstacles and non-obstacles from terrain images because the VI-based networks are deep enough to have good feature extraction capabilities to solve this problem. Thus, even in cases where the single-step prediction accuracy is not very high, the model is more likely to plan successful paths, due to the small probability of collision with obstacles. Overall, the dVIN significantly outperforms the baseline models, with natural images as input, and generalizes well to large-size domains, thanks to the two-stage training strategy.

## 5. Conclusions

In this work, we propose a novel end-to-end planning model, named the double value iteration network (dVIN). When using terrain images of the planets’ surface as input, the dVIN can output safe and energy-efficient paths through the explicit planning computation, to help rovers perform better in their exploration missions. We introduce a weighted double estimator approach to the VIN, with one estimator determining the maximizing action and the other providing the estimate of its value. We then use the weighted mean of the two values obtained to approximate the maximum expected value of the next iteration, intending to reduce the overestimation and result in more stable and reliable learning. We compare the dVIN with three baseline methods, including VIN, SVIN, and DB-CNN, on path planning tasks of grid-world domains and a data set generated from lunar terrain images. The experimental results show that the dVIN significantly outperforms the other VI-based models and generalizes better on the test set, in comparison to the CNN-based model. In addition, we design a simple, yet effective, two-stage training strategy, which involves full training on small-size maps and fine-tuning on a few large-size maps. When trained with this technique, the VIN and SVIN can achieve similar performance as the DB-CNN as the size of environments grows, and the dVIN performs much better than the baseline methods. By pre-training on the 32×32 lunar domain, our dVIN achieves an 89.82% success rate on the 128×128 domain, outperforming the VIN by 30.03% and SVIN by 26.59%, while using less computing resources than training from scratch.

## Figures and Tables

**Table 1:** Performance on 28×28 grid-world domain.

Training Strategies	Methods	Pred. Loss	Succ. Rate	Traj. Diff.
step LR	VIN	0.129	89.31%	0.451
	SVIN	0.121	89.72%	0.405
	DB-CNN	0.079	97.78%	0.402
	dVIN (ours)	**0.050**	**99.45%**	**0.120**
1cycle LR	VIN	0.121	92.01%	0.454
	SVIN	0.103	94.50%	0.330
	DB-CNN	0.076	98.57%	0.402
	dVIN (ours)	**0.033**	**99.93%**	**0.032**

**Table 2:** Performance on 64×64 and 128×128 grid-world domain. All models were only trained in 28×28 domains and directly tested in large-size domains.

Methods	64×64		128×128	
	Succ. Rate	Traj. Diff.	Succ. Rate	Traj. Diff.
VIN	69.96%	1.623	35.10%	2.502
SVIN	74.53%	1.289	37.04%	2.025
DB-CNN*	37.02%	2.654	6.68%	1.361
dVIN (ours)	**87.65%**	**0.169**	**60.90%**	**0.183**

**Table 3:** Performance on 64×64 and 128×128 grid-world domain. All models were first trained in 28×28 domains and then fine-tuned with a few large-size instances.

Methods	64×64			128×128		
	Pred. Loss	Succ. Rate	Traj. Diff.	Pred. Loss	Succ. Rate	Traj. Diff.
VIN	0.178	70.56%	1.450	0.203	49.01%	2.996
SVIN	0.170	76.75%	1.336	0.184	53.56%	2.207
DB-CNN*	0.178	55.70%	3.374	0.348	16.74%	6.203
dVIN(*ours*)	**0.037**	**98.85%**	**0.103**	**0.062**	**88.66%**	**0.822**

**Table 4:** Performance on lunar domain. For all domain sizes, the dVIN significantly outperform other VI-based models. Note that the performance gap increases dramatically with problem size.

Methods	32×32		64×64		128×128	
	Succ. Rate	Traj. Diff.	Succ. Rate	Traj. Diff.	Succ. Rate	Traj. Diff.
VIN	95.17%	0.236	78.57%	1.255	59.79%	2.234
SVIN	96.43%	0.193	80.65%	1.131	63.23%	2.173
dVIN(ours)	**99.85%**	**0.076**	**98.19%**	**0.138**	**89.82%**	**0.478**

## Data Availability

No new data were created or analyzed in this study. Data sharing is not applicable to this article.
